# Editorial: Improving our understanding of the management and pathogenesis of rare and neglected tumors of the central and peripheral nervous system

**DOI:** 10.3389/fonc.2023.1162728

**Published:** 2023-03-17

**Authors:** Ignazio G. Vetrano, Pierpaolo Alongi, Laura Gatti

**Affiliations:** ^1^ Department of Neurosurgery, Fondazione IRCCS Istituto Neurologico Carlo Besta, Milan, Italy; ^2^ Department of Biomedical Sciences for Health, Università degli Studi di Milano, Milan, Italy; ^3^ Nuclear Medicine Unit, A.R.N.A.S Ospedale Civico Di Cristina e Benfratelli, Palermo, Italy; ^4^ Neurobiology Laboratory, Fondazione IRCCS Istituto Neurologico Carlo Besta, Milan, Italy

**Keywords:** DIPG (diffuse infiltrative pontine gliomas), intracranial lymphomas, MPNST (malignant peripheral nerve sheath tumors), pleomorphic xanthoastrocytomas, sodium fluorescein (SF), solitary fibrous tumor (SFT), astroblastoma, peripheral nerve sheath tumors

The Frontiers Research Topic titled “*Improving our Understanding of the Management and Pathogenesis of Rare and Neglected Tumors of the Central and Peripheral Nervous System*” includes 22 articles published from May 2022 to February 2023. This collection comprises 13 original research articles, 7 case reports, and 2 reviews, with the common aim to collect information about clinical and surgical management, new diagnostic techniques (pre-, post-, and intraoperative), innovative therapeutic therapies, as well as preclinical studies based on genetic, cellular, molecular, or omic approaches, dedicated to rare tumors of the central and peripheral nervous system in both pediatric and adult populations. Although uncommon compared to other tumors, primary Central Nervous System (CNS) and Peripheral Nervous System (PNS) cancers can cause severe morbidity and mortality across all populations. Despite the efforts and care improvement dedicated to the most prevalent brain and spine tumors (such as glioblastomas, metastases, or meningiomas), some of these diseases still lack defined management plans or specific preclinical assessment of their cellular/molecular features, thus affecting patients’ management.

While CNS tumors can now be much more precisely characterized than a few years ago, the translation of this increased knowledge into more effective treatments is still seriously lagging behind, in particular considering specific populations such as children or the elderly. This is particularly true for pediatric CNS tumors. In a large series of 80 children, Zhang et al. confirmed that Diffuse Intrisic Pontine Glioma (DIPG, included in Diffuse Midline Gliomas, H3K27 altered) display a broad spectrum of clinical and imaging features; whereas surgery could play a role in addition to adjuvant therapies, H3K27 alteration was the independent prognostic influencing factor. Falco et al. analyzed the role of intraoperative fluorescent dyes in the surgical management of pleomorphic xanthoastrocytoma (PXA), a rare brain tumor most commonly affecting children and young adults. Surgical resection is the mainstay of treatment, and the extent of resection is associated with improved survival. Among the twelve patients included, comprising three pediatric patients, sodium fluorescein helped distinguish tumors from viable tissue in all cases. Their data suggest a role in improving the extent of resection during surgery of PXA. Merchant et al. presented a case of Oligodendroglioma IDH-mutant and 1p/19q-codeleted associated with a germline mutation in PMS2 like Lynch Syndrome, to evaluate the role of germline PMS2 mutations in gliomas, and highlighted the importance of genetic testing in neuro-oncology. Frederico et al. elegantly analyzed the heterogenicity among adult patients harboring MN1-altered CNS tumors. These uncommon lesions were recently added to the 2021 WHO classification under the name Astroblastoma, MN1-altered. Thought to occur most commonly in children and predominantly in females, MN1-altered CNS tumors are associated with typical (whereas not pathognomonic) histological patterns, with a distinct DNA methylation profile and recurrent fusions implicating the MN1 (meningioma 1) gene. The authors emphasize the diagnostic challenges, considering that most cases with morphological features of astroblastoma (but not all) show these molecular features, whereas not all tumors with MN1 fusions show astroblastoma morphology. From a clinical point of view, there is significant variability in reported outcomes: multiple recurrences are frequent, despite multimodality treatments. Additionally, the authors propose a standardized model for patient-reported outcomes. Pavlova et al. evaluated, in the preclinical setting, the effect of radiation therapy on patient-derived glioblastoma cells to evaluate the contribution of homologous recombination and nonhomologous end in DNA break repair after exposure to different radiation doses.

Solitary fibrous tumor (SFT), previously known as hemangiopericytoma or SFT/hemangiopericytoma, is a rare intracranial malignancy thought to originate from pericyte cells lining the capillary walls. These tumors represent less than 1% of intracranial tumors and are frequently mistaken for meningiomas on imaging. Unlike most meningiomas, however, SFT has a propensity for local recurrence and extracranial metastasis after resection. Surgery usually represents the pivotal therapeutic point, but the role of adjuvant treatment is often unclear. Three papers analyzed the impact of radiation therapy in the management of SFT. Liu et al. evaluated 38 patients with SFT, confirming that patients with high WHO-grade SFT have an impaired PFS and reduced OS, which appears even more negatively affected by a subtotal resection. Postoperative radiotherapy increases the local control rate in patients with WHO grade 3 tumors. Gou et al. focused on the role of postoperative stereotactic radiosurgery or intensity-modulated radiotherapy; this second option seems to increase disease-free survival compared to radiosurgery. Finally, Allen et al. proposed for the first time the use of a mixed-modality, multi-fraction radiosurgery technique to treat recurrent SFT, to maximize radiation dose to the targets while minimizing complication risk after resection.

Three papers analyzed different aspects of intracranial lymphomas, considering that intracranial lymphomas can mimic different brain tumors. Yang et al. evaluated the impact of risk factors such as age, Ann Arbor stage, and treatment in the prognosis of primary intracranial malignant lymphomas. Cheng et al. evaluated a series of primary ventricular lymphomas, an extremely rare and frequently misdiagnosed disease, with high mortality (median survival time of 15 months). Wang et al. evaluated the case of a suspected pituitary apoplexy, in which the diagnosis was lymphoblastic lymphoma derived from B-cells.

Other papers investigated different CNS tumors, included within a wide spectrum of embryonal origin and clinical behaviors. Li et al. evaluated a *de novo* mutation in von Hippel–Lindau (VHL) syndrome, performing whole-exon gene analysis to improve the understanding of the diagnosis. Early recognition and treatment of VHL syndrome can also be available with genetic testing technology in case of a negative familial history. Strengthening the understanding of this complex genetic disease and improving the diagnostic rate of VHL syndrome is helpful for personalized treatments. Xu et al. assessed clinicopathological characteristics, prognostic factors, and outcomes in a series of 17 children harboring embryonal tumors with multilayered rosettes (C19MC-altered or not elsewhere classified). They confirmed the aggressive behaviors of such tumors: for patients receiving chemotherapy, the median overall survival time was 7.4 months, while those who did not receive chemotherapy was 1.2 months. Children older than four years tend to have a higher rate of metastasis.

Kim et al. evaluated the impact of Gamma-Knife radiosurgery for vestibular schwannomas in neurofibromatosis type 2 patients. Longitudinal volumetric analyses showed that most treated lesions showed effective tumor control up to 85% at 60 months, whereas unirradiated lesions progressed with a relative volume increase of 14.0% (7.8-27.0) per year during the observation period. However, 29% of cases showed pseudoprogression with significant volume expansion in the early follow-up period, which practically reduced the tumor control rate to 57% at 24 months, and the short-term effects of this treatment are not highly advantageous in terms of hearing preservation. Therefore, careful patients selection is necessary for such treatment.

Fang et al. presented a gene expression evaluation of adamantinomatous craniopharyngioma (ACP), an epithelial tumor arising from Rathke’s pouch remains. They screened for differentially expressed genes (DEGs) to identify key signaling pathways. Hierarchical clustering showed that the DEGs could precisely distinguish the ACP group from the control group, suggesting that E-cadherin (*CDH1*) may play a relevant role in the pathways in cancer signaling pathway that regulates ACP development, and it could be a target suitable for precision medicine.

Feng et al. conducted a Surveillance, Epidemiology, and End Results population-based study for the elderly with malignant meningiomas, a rare form of a relatively common CNS tumor, with poor survival. The multivariable analysis among this specific population revealed that surgical resection is recommended for elderly patients with malignant meningiomas (if surgery is not contraindicated for systemic, patient-specific factors). However, gross-total resection does not significatively impact patients’ survival, compared to subtotal resection in older patients. Also, Serratrice et al. evaluated meningiomas but focused on spinal forms. They reviewed the fundamental epidemiological and clinical aspects of spinal meningiomas, their histological and genetic characteristics, and their management, including updated surgical advancements.

The remainder of the accepted manuscripts studied PNS tumors, from preclinical studies to clinical and surgical points of view. The special issue comprises six papers based on Peripheral Nerve Sheath Tumors (PNST). Gu et al. performed a preclinical assessment of MEK inhibitors for Malignant PNST (MPNSTs), rare soft-tissue sarcomas refractory to standard therapies. The majority of MPNSTs show inactivation of NF1 and upregulation of RAS/RAF/MEK/ERK signaling pathways. The authors evaluated different MEK inhibitors in terms of efficacy, safety, and mechanism of adaptive response in the case of MPNSTs. Using a tissue microarray, they identified p-ERK as a biomarker for predicting the prognosis of MPNST patients as well as an effective therapeutic target. Trametinib consequentially appeared as the most potent MEK inhibitor for treating MPNSTs. Globally, reduced reactivation of the MAPK pathway and compensatory activation of the parallel pathways contributed to better efficacy.

Imaging differentiation among benign and malignant PNST is pivotal in Neurofibromatosis type 1. Liu et al. reviewed different non-invasive image-based diagnostic common findings. Moreover, the addition of novel technologies like radiogenomics can introduce future perspectives that ultimately can contribute to the radiology image-based clinical screening of MPNST in NF1 patients. Bonomo et al. provided a new case of sporadic spinal psammomatous malignant melanotic Nerve Sheath Tumor (SSP-MMNST), a rare subgroup of PNST arising along the spine, with only a few reports described. The literature review identified 21 eligible studies assessing 23 patients, with a mean onset age of 41 years and a slight male gender predominance. In all cases, resection is the treatment of choice in all amenable cases, followed in selected cases with residual tumors by adjuvant radiotherapy or chemotherapy.

Nevertheless, the metastatic and recurrence rates were 31.58% and 36.8%, respectively. Wei et al. focused on the differential diagnosis of tuberous sclerosis and neurofibromatosis type 1. The treatments for the two diseases vary significantly, and misdiagnoses can seriously threaten the patient’s health. Finally, Nazzi et al. assessed the role of sodium fluorescein in PNST, evaluating a comprehensive series of 142 cases submitted to fluorescein-guided surgery. In fact, surgery is the mainstay of treatment for PNST, but sometimes distinguishing between intact functional nerve and the fibers from whence the PNST arose may not always be easy to perform, constituting the most relevant risk factor in determining a worsening neurological condition. Intraoperative fluorescein characteristics and postoperative neurological and radiological outcomes were analyzed and compared with a historical series. Bright fluorescence was present in all schwannomas and neurofibromas, although with a less homogeneous pattern, whereas it was significantly less evident for MPNST. The authors concluded that SF is a valuable method for safe fluorescence-guided resection of PNST and mimicking lesions, with a positive effect mainly in plexiform neurofibromas, suggesting a possible role in improving the functional and oncological outcome of these lesions.

In summary, the special issue provides great information on different, rare central and peripheral nervous system tumors. The editors hope that this collection of knowledge can be exploited to help improve knowledge and research on neuro-oncology.

## Author contributions

All authors listed have made a substantial, direct, and intellectual contribution to the work and approved it for publication.

